# A blood test-based nomogram to predict the progression-free survival of patients with intrahepatic cholangiocarcinoma after surgical resection

**DOI:** 10.3389/fonc.2025.1507602

**Published:** 2025-06-09

**Authors:** Lirong Peng, Yang Shi, Shuang Yang, Cunyan Li

**Affiliations:** 1 Department of Clinical Laboratory, The First Affiliated Hospital of Hunan Normal University, Changsha, China; 2 Department of Research and Development, Human Stem Cell National Engineering Research Center, Changsha, China

**Keywords:** intrahepatic cholangiocarcinoma, progression free survival, nomogram, blood biomarkers, prognostic model

## Abstract

**Background:**

Intrahepatic cholangiocarcinoma (ICC) is a highly aggressive malignancy with poor prognosis, and there is currently a lack of effective prognostic prediction models. The aim of this study was to develop a novel nomogram model based on blood tests for predicting predictors of progression free survival (PFS) in ICC patients.

**Methods:**

A total of 99 ICC patients (70 for training, 29 for validation) were included in this study. Hematological indices and clinicopathological data were collected from ICC patients undergoing surgical resection. The independent predictors of PFS were screened by univariate and multivariate Cox regression analysis, and a nomogram model was constructed. The calibration curve was used to evaluate the consistency between the observed results and the predicted probability, and the model discrimination was evaluated by receiver operating characteristic curve (ROC). According to the risk score calculated by the constructed nomogram, patients were divided into high-risk and low-risk groups, and the predictive performance of nomogram was further tested by Kaplan Meier.

**Results:**

The median follow-up time of this study was 7.8 months (range: 1 ~ 69 months). We found that pathological differentiation, CA19-9, neutrophil-to-lymphocyte ratio (NLR) and after-treatment Monocyte count (MON)/before-treatment MON (tMON) were independent factors affecting the PFS of postoperative ICC patients. Based on risk factors, a nomogram prediction model was constructed. ROC analysis revealed that the area under the curve (AUC) of the nomogram for predicting PFS was higher than the AJCC-TNM staging system(*P*<0.05). The calibration curve and decision curve analysis (DCA) showed that the nomogram had high prognostic accuracy and clinical applicability. The risk score calculated by nomogram could divide ICC patients into high-risk and low-risk groups. The median PFS of the high-risk group was significantly shorter than that of the low-risk group (*P <*0.05).

**Conclusion:**

The nomogram can serve as a valuable supplementary tool for predicting PFS in ICC patients after initial surgical resection. Its performance is better than the traditional TNM staging system. The model provides clinicians with an individualized prognostic assessment tool by integrating easily available blood markers, which is helpful to optimize postoperative monitoring and adjuvant treatment strategies.

## Introduction

Cholangiocarcinoma is the second most common type of liver cancer after hepatocellular carcinoma, accounting for 10-15% of all primary malignant tumors of the liver. Over the past decade, the incidence and mortality rates of cholangiocarcinoma have been increasing globally (1). Although radical surgical resection is still the only potential cure, about 65% of patients have recurrence and metastasis within two years after surgery, resulting in a 5-year overall survival rate of less than 30% (2). This critical situation underscores the importance of accurate progression-free survival (PFS) prediction for individualized postoperative management.

Current clinical practice predominantly relies on the AJCC 8th edition TNM staging system for prognostic assessment. However, this anatomy-based classification system demonstrates significant limitations, with only modest predictive accuracy (C-index ≈0.60-0.65), making it inadequate for guiding individualized treatment strategies (3, 4). Although recent studies have attempted to develop predictive models by incorporating genomic/proteomic signatures and radiomic features, their clinical translation remains limited due to either high testing costs or the requirement for tissue specimens that preclude dynamic monitoring (5).

Serum biomarkers represent a promising avenue for refining prognostic tools owing to their noninvasive nature and capacity for repeated measurements. Emerging evidence suggests that individual blood biomarkers—including CA19-9– – (6), systemic inflammation indices (e.g., NLR, PLR), and liver function markers (e.g., ALBI score)—may correlate with ICC progression (7). However, a multidimensional blood parameter-based model for predicting PFS remains to be established. In this study, we aim to establish a blood-based PFS prediction nomogram for ICC patients by analyzing the baseline characteristics of ICC patients and the levels and changes of blood markers before and after treatment.

## Patients and methods

### Patients

This study retrospectively included a total of 99 patients with ICC who underwent initial surgical resection treatment at Hunan Provincial People’s Hospital between January 2020 and December 2023. The inclusion criteria for patient selection were as follows: 1) Pathological confirmation of ICC; 2) No prior anti-tumor treatment before pathological diagnosis; 3) ECOG performance status score of 0 or 1. The exclusion criteria consisted of the following: 1) Prior anticancer treatment before admission; 2) History of other malignancies; 3) Patients with metastatic bile duct cancer; 4) Incomplete clinical data and incomplete laboratory examination data before and after treatment. This retrospective study was approved by the Ethics Committee of Hunan Provincial People’s Hospital, and informed consent requirements were waived.

### Laboratory examination

The laboratory test indicators of liver function-related tests including total protein (TP), albumin (ALB), globulin (GLB), total bilirubin (TBIL), alanine aminotransferase (ALT), aspartate aminotransferase (AST), total bile acids (TBA) were measured using a Hitachi LABOSPECT 008 AS fully automated biochemical analyzer. Blood routine examinations: neutrophil (NEU) count, lymphocyte (LYM) count, MON count, red blood cell (RBC) and platelet (PLT) count were measured using the Sysmex XN-9000 automated hematology analyzer. Coagulation-related tests: prothrombin time (PT), international normalized ratio of prothrombin time (INR), activated partial thromboplastin time (APTT), thrombin time (TT), fibrinogen (FIB) were analyzed using the Sysmex CS-2500 automated coagulation analyzer. Alpha-fetoprotein (AFP) and CA19–9 were quantitatively measured using the Roche Cobas e601 electrochemiluminescence immunoassay analyze. The calculated values in this study were determined using the following formulas: NLR = neutrophil count/lymphocyte count, tMON = after-treatment MON/before-treatment MON.

### Statistical analysis

Categorical variables were presented as the number of cases (percentage), and chi square test was used for difference analysis. All continuous variables were first evaluated for their normal distribution characteristics by Shapiro Wilk test. For variables that conform to normal distribution, we use the mean ± standard deviation (mean ± SD) to describe; For non-normally distributed variables, the median and interquartile range are used. Mann-Whitney U test was used for comparison. All potential predictive factors (including clinicopathological characteristics and blood test indicators) were first screened by univariate Cox regression analysis, and variables significantly associated with PFS (p<0.05) were selected as candidate factors. These candidate factors were then entered into the multivariate Cox regression analysis, and the variables with p<0.05 were finally determined as independent predictors. Based on the results of multivariate Cox regression analysis, a nomogram was generated using the CPH function of R package RMS. The prediction performance of the model was evaluated by ROC, calibration curve and DCA decision curve. Finally, the risk score of each patient was calculated according to the nomogram, and the patients were divided into high-risk group and low-risk group according to the score. Kaplan Meier method was used to compare PFS between high-risk group and low-risk group. P<0.05 means the difference is statistically significant. SPSS 26.0 statistical software and R language (4.4.2) software were used for statistical analysis and nomogram drawing.

## Results

### Patient characteristics

A total of 99 patients (60 males and 39 females) in this study were randomly divided into two groups (training set, n=70 cases; validation set, n=29 cases). The clinical demographics data of the training and validation sets are presented in **Table 1**. The mean age of the patients was 58.9 ± 12.1 years, and the tumor diameter was 4.4 (2.9-6.3) cm. Based on radiological data evaluation, 40 out of the 99 patients had portal vein tumor thrombus (PVTT). The median PFS for ICC patients in this study was 7.8 months (ranging from 1 to 69 months), and the half-year, one year, and two years PFS rates were 58.6%, 32.3%, and 13.1%, respectively.

**Table 1:** Baseline characteristics of enrolled patients.

Characteristics	Baseline	Training set	Validation set	*P* value
Age (y)	58.9±12.1	57.4±12.3	62.4±11.3	0.063
Gender (n, %)				1.000
Male	60 (61.6)	42 (60.0)	18 (62.1)	
Female	39 (39.4)	28 (40.0)	11 (37.9)	
Differentiation (n, %)				0.548
Poor	20 (20.2)	16 (22.9)	4 (13.8)	
Moderate and poor	32 (32.3)	23 (32.8)	9 (31.0)	
Moderate	38 (38.4)	24 (34.3)	14 (48.3)	
Well	9 (9.1)	7 (10.0)	2 (6.9)	
Tumor diameter, cm	4.4 (2.9-6.3)	4.9 (3.2-6.7)	3.5 (2.5-5.4)	0.070
PVTT (n, %)				1.0
Positive	40 (40.4)	28 (40.0)	12 (41.4)	
Negative	59 (59.6)	42 (60.0)	17 (58.6)	
TNM stage (n, %)				0.646
I	28 (28.3)	21 (30.0)	7 (24.1)	
II	27 (27.3)	17 (24.3)	10 (34.5)	
III	18 (18.2)	12 (17.1)	6 (20.7)	
IV	26 (26.2)	20 (28.6)	6 (20.7)	
PT (s)	10.8±1.1	10.7±1.1	11.0±1.0	0.168
INR	0.9 (0.9-1.0)	0.9 (0.9-1.0)	1.0 (0.9-1.0)	0.155
APTT (s)	26.3 (24.9-27.5)	26.2 (24.9-27.1)	27.2 (24.0-28.3)	0.244
TT (s)	17.7 (16.8-18.8)	17.7 (16.7-18.9)	17.8 (17.2-18.8)	0.643
FIB (g/L)	3.7 (3.1-4.2)	3.7 (3.2-4.3)	3.5 (2.6-4.1)	0.283
AFP (ng/mL)	4.2 (2.5-8.0)	4.2 (2.7-9.6)	4.2 (2.4-5.9)	0.405
CA19-9 (U/mL)	73.6 (25.0-271.3)	64.3 (18.1-208.7)	86.9 (42.1-474.4)	0.070

### Levels of blood parameters before and after surgical treatment

We analyzed the levels and changes of blood biomarkers in ICC patients before and after surgical treatment. The results showed that most blood markers changed significantly one week after treatment compared to before treatment. Levels of TP, ALB, GLB, TBIL, AST, TBA and RBC decreased significantly after one week of treatment, while levels of NEU, MON and NLR increased significantly. Changes in ALT, LYM and PLT before and after treatment were not significant. Details of blood marker levels before and after treatment are described in **Table 2**.

**Table 2:** Levels of blood parameters before and after treatment.

Characteristics	Before treatment	1 week after treatment	*P* value
TP	64.78 (60.40-68.52)	58.50 (52.40-65.30)	<0.001
ALB	38.91±5.09	34.40±5.00	<0.001
GLB	26.14±4.78	24.41±6.95	0.007
TBIL	19.00 (13.90-89.04)	19.94 (12.40-57.11)	0.002
ALT	55.40 (25.40-127.7)	65.60 (38.20-105.90)	0.799
AST	54.30 (30.20-77.24)	38.10 (27.44-68.00)	0.001
TBA	7.33 (4.50-86.40)	5.60 (3.34-11.64)	<0.001
NEU	4.35 (3.21-5.91)	5.25 (3.66-7.46)	0.015
LYM	1.42±0.51	1.34±0.61	0.086
MON	0.56 (0.43-0.70)	0.69 (0.49-0.98)	<0.001
RBC	4.39±0.65	3.82±0.73	<0.001
PLT	212.00 (172.00-267.00)	211.00 (155.0-273.0)	0.500
NLR	3.34 (2.64-4.41)	4.18 (2.70-7.11)	<0.001

### Screening for prognostic factors related to PFS in ICC patients

Univariate Cox regression analysis was used to explore the correlation between PFS and each clinical factor and blood marker in the training set. The results revealed that lower tumor pathological differentiation correlated with greater patient risk, and higher TNM staging was associated with increased patient risk. The blood markers such as CA19-9, GLB, NLR, after-treatment TP (aTP), after-treatment GLB (aGLB), trend NEU (tNEU) and tMON were identified as risk factors, while trend ALB (tALB) were identified as protective factors (**Table 3**).

**Table 3:** Univariate and multivariate Cox analyses for PFS of ICC patients.

Variables	Univariate Analysis		Multivariate Analysis	
	P	HR (95% CI)	P	HR (95% CI)
TNM	0.004	1.503 (1.136-1.989)		
Differentiation	<0.001	0.348 (0.229-0.528)	<0.001	0.306 (0.177-0.530)
CA199	0.015	1.001 (1.000-1.001)	0.028	1.001 (1.000-1.002)
GLB	0.022	1.089 (1.013-1.171)		
NLR	<0.001	1.357 (1.144-1.610)	0.041	1.224 (1.008-1.486)
aTP	0.001	1.072 (1.028-1.119)		
aGLB	<0.001	1.090 (1.035-1.147)		
tALB	0.003	0.297 (0.135-0.656)		
tNEU	0.018	2.084 (1.137-3.820)		
tMON	<0.001	3.190 (1.687-6.033)	0.015	2.852 (1.226-6.634)

aTP, after-treatment TP; aGLB, after-treatment GLB; tALB, trend (after-treatment/before-treatment) ALB; tNEU, trend NEU; tMON, trend MON.

### Establishment of prognostic nomogram for evaluating PFS in ICC patients

Multivariable Cox regression analysis was performed on the factors selected through univariate Cox regression analysis in the training set, including clinical factors and candidate blood indicators. Among all clinical factors, pathological differentiation was the only statistically significant independent prognostic factor. Within the hematological indicators, CA19-9, NLR and tMON were identified as independent prognostic factors for ICC patients’ PFS (**Table 3**).

**Figure 1:**
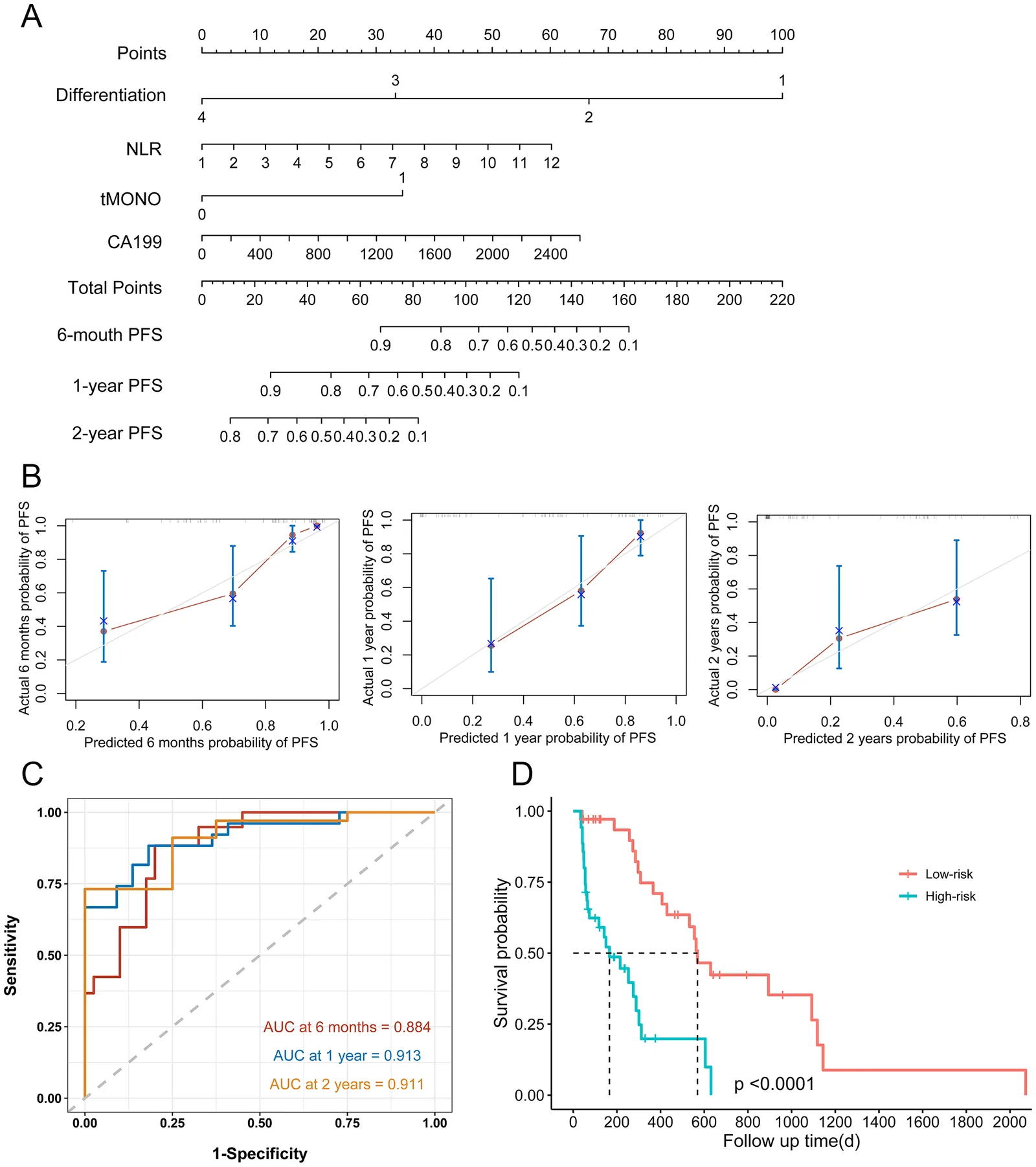
Nomogram for PFS in patients with ICC after surgical resection. **(A)** Nomogram model for half-year, 1-year, and 2-year PFS in the training set. **(B)** Calibration curves plots of nomogram for predicting half-year, 1-year, and 2-year probability of PFS in ICC patients after surgical resection in the training set **(C)** ROC curves of the nomogram in the training set. **(D)** Prognostic assessment and risk stratification of developed nomogram model in the training set.

A nomogram was established based on the results of the multivariable Cox regression analysis in the training set (**Figure 1A**). Calibration plots were generated to assess the agreement between predicted and actual PFS rates at half-year, one-year, and two-year intervals. The calibration curves for these intervals showed substantial overlap with the standard curve (**Figure 1B**). This indicates that the nomogram has good predictive efficacy. The performance of the nomogram was further evaluated using ROC curves. The AUCs of the nomogram for predicting PFS at six months, one year, and two years were 0.884, 0.913, and 0.911, respectively (**Figure 1C**). The nomogram showed significantly better performance compared to the commonly used AJCC TNM staging system, for the AJCC TNM system, the AUCs were 0.791, 0.704, 0.581 at the corresponding time points (Supplementary **Figure 1A**). Based on the established nomogram, scores were calculated for each patient, and further stratification divided patients into low-risk (below median score of 85) and high-risk groups (above median score). The median PFS was 14.3 months (429 days) in the low-risk group and 4 months (121 days) in the high-risk group (*P* < 0.0001, **Figure 1D**)

### Validation the prognostic nomogram model

**Figure 2:**
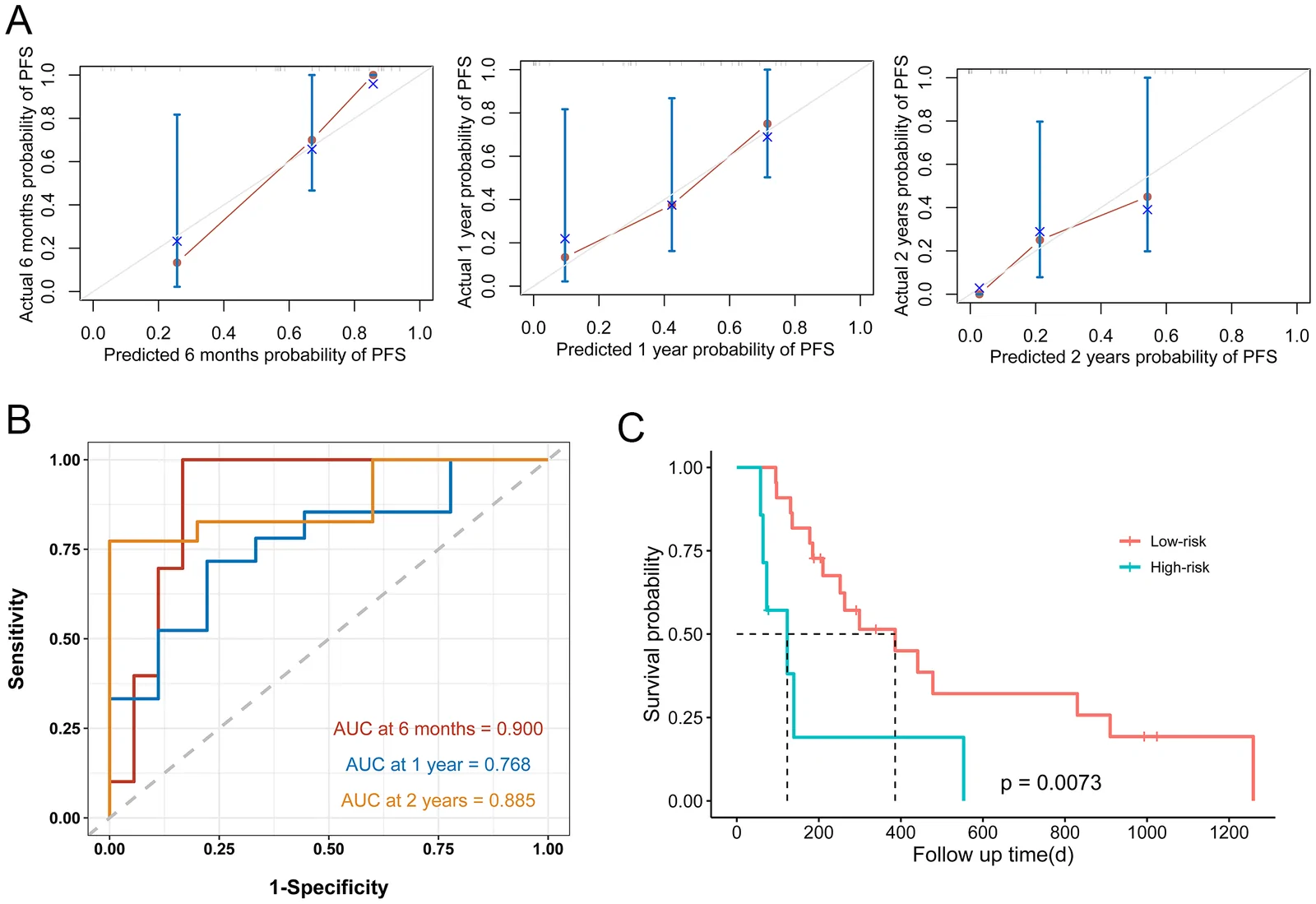
Nomogram model performance in the validation set. **(A)** Calibration curves plots of nomogram for predicting half-year, 1-year, and 2-year probability of PFS in ICC patients after surgical resection in the validation set **(B)** ROC curves of the nomogram in the validation set. **(C)** Prognostic assessment and risk stratification of developed nomogram model in the validation set.

To validate the nomogram model, we introduced a validation set. The calibration plot demonstrated moderate agreement between predicted and observed outcomes (**Figure 2A**). For predicting progression-free survival (PFS), the nomogram achieved AUCs of 0.900, 0.768, and 0.885 at six months, one year, and two years, respectively, in the validation cohort (**Figure 2B**). For the AJCC TNM system, the AUCs were 0.591, 0.649, 0.730 at the corresponding time points (**Supplementary Figure 1B**). Using the same cutoff value as in the training set, we stratified the validation cohort into low- and high-risk groups. The median PFS was significantly longer in the low-risk group (14.3 months) than in the high-risk group (4 months; *P* = 0.0073), confirming robust risk stratification (**Figure 2C**).

### DCA for clinical utility of the nomogram

**Figure 3:**
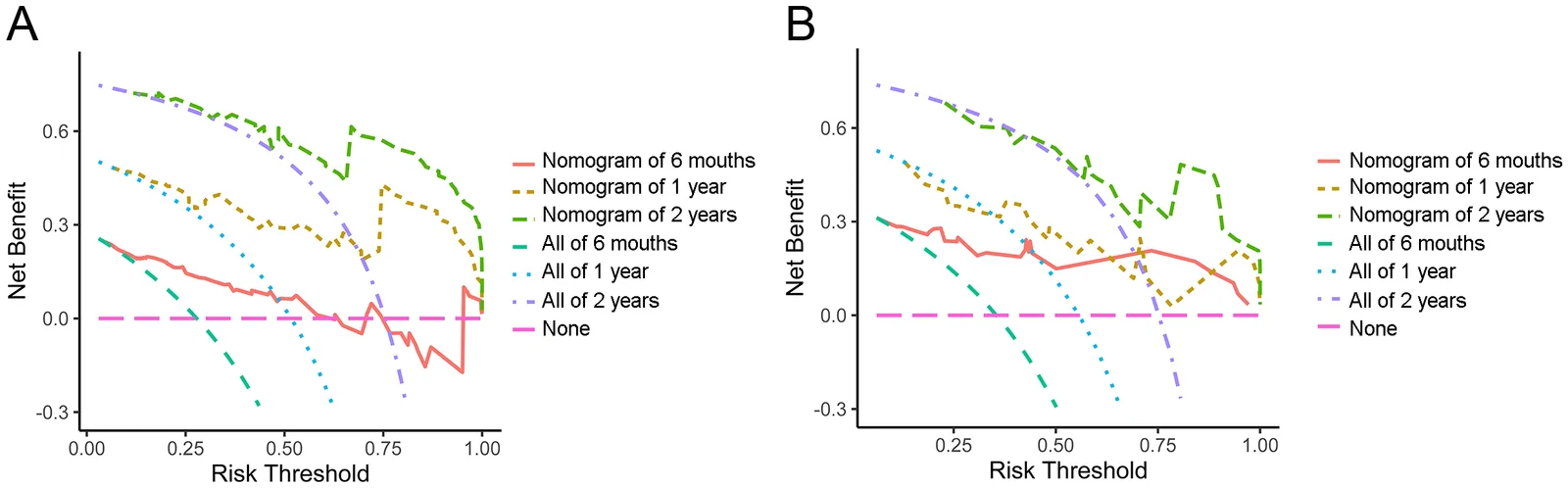
Decision curve analysis of half-year, 1-year and 2-year PFS in the training set **(A)** and validation set **(B)**.

The DCA curve, which is employed for the purpose of evaluating the clinical utility of the nomogram, is illustrated in **Figure 3**. The DCA analysis demonstrated that the nomogram model has the potential to enhance net benefits and exhibited a broader range of threshold probabilities in the prediction of PFS in ICC patients.

## Discussion

ICC is characterized by a high overall malignancy and a high recurrence rate, leading to poor prognosis for patients. Even after radical resection, the 5-year survival rate remains below 20% (8).Although various treatment modalities have shown differences in OS, the overall OS remains limited (9). It has been reported that the average weighted OS for ICC patients receiving local treatment is only 15.7 months, while those receiving first-line treatment with concurrent systemic chemotherapy have an OS of 25.2 months (10). Although multiple treatment options are currently available for intrahepatic cholangiocarcinoma (ICC) patients, surgical resection remains the only potentially curative approach (11). Predictive models can assist clinicians in evaluating disease progression risk and prognosis, thereby facilitating personalized treatment strategies. Furthermore, by predicting patient outcomes, these models enable prioritization of more aggressive therapies for high-risk patients while avoiding overtreatment of low-risk individuals.

In this study, a nomogram model for predicting postoperative PFS of ICC patients was constructed by integrating clinicopathological parameters and dynamic hematological biomarkers. The nomogram showed significantly better performance compared to the commonly used AJCC TNM staging system, with AUCs of 0.884, 0.913, and 0.911 in the training set and 0.900, 0.768 and 0.885 in the validation set at six months, one year, and two years, respectively, while the AUCs for the AJCC TNM system in the training set were 0.791, 0.704, 0.581, and 0.591, 0.649, 0.730 in the validation set at the corresponding time points. Recent years have witnessed remarkable advancements in cancer diagnosis and prognosis, driven by technological innovations and scientific breakthroughs. An array of novel methodologies has emerged for prognostic evaluation, including liquid biopsy (encompassing ctDNA/CTC analysis) (12), multi-omics profiling (spanning genomic, transcriptomic, and epigenomic dimensions) (13), radiomics (leveraging AI-based imaging feature extraction) (14), and comprehensive tumor microenvironment characterization (e.g., Immunoscore systems) (15). However, research on predictive models for postoperative outcomes in intrahepatic cholangiocarcinoma (ICC) remains limited (11, 16, 17). Existing studies have incorporated various factors including immune-inflammatory indices (7, 18), gene expression and modification profiles (19, 20), treatment modalities (21, 22), pathological parameters and imaging characteristics (23, 24). While current models demonstrate varying degrees of prognostic capability for ICC patients, they predominantly focus on overall survival while inadequately addressing the dynamic disease progression. In this study, we incorporated not only baseline clinicopathological parameters but also serial hematological biomarkers measured before treatment and two weeks postoperatively, with their dynamic changes included in the analysis to highlight the predictive value of longitudinal monitoring. Through multivariate Cox regression analysis, we identified pathological differentiation, CA19–9 levels, neutrophil-to-lymphocyte ratio (NLR), and temporal monocyte counts (tMONO) as independent prognostic factors for progression-free survival (PFS) in ICC patients following resection. Pathological differentiation and AJCC TNM classification are among the main factors affecting the development and prognosis of ICC (25, 26). Our results showed that the lower the degree of differentiation, the higher the risk of patients (HR:0.306, 95% CI:0.177~0.530, *P*<0.001), which is consistent with mainstream reports (27, 28).Hematological indicators have unparalleled advantages for tumor follow-up due to their ease of acquisition, ability for repeated sampling, and potential for early detection before imaging changes occur. Serum tumor marker CA19–9 is the most widely used diagnostic and prognostic indicator for ICC patients (29, 30). Consistent with the findings of Sanchez L et al. our study confirms that CA19–9 serves as an independent prognostic factor in patients with ICC– (1). Additionally, The NLR and platelet-to-lymphocyte ratio (PLR) serve as indicators of systemic inflammatory status, where chronic inflammation promotes immunosuppression and angiogenesis within the tumor microenvironment (31, 32). Our study validated NLR as an independent prognostic factor, highlighting the role of inflammatory markers in hepatobiliary malignancies. To evaluate the prognostic value of dynamic parameter changes following surgery, we analyzed postoperative hematological trends and identified the preoperative-to-postoperative monocyte ratio (tMON) as an independent prognostic factor. To our knowledge, this is the first study to validate tMON for ICC outcome prediction. The incorporation of this novel biomarker may enable earlier risk stratification and optimized patient surveillance.

It should be noted, however, that the model established in this study is not without limitations. Firstly, the optimal endpoint for follow-up should be OS; Nevertheless, given the brief period of patient enrolment, PFS was selected as a surrogate endpoint. Secondly, this is a single center, retrospective study. It would be beneficial to validate the results further with data from other centers. Finally, due to data accessibility constraints, our model did not account for potential influences of postoperative adjuvant therapies on PFS outcomes.

## Conclusion

This study developed a novel nomogram model for predicting postoperative progression-free survival (PFS) in patients with intrahepatic cholangiocarcinoma (ICC). The nomogram demonstrated excellent discriminative ability and calibration in both training and validation cohorts. Compared with the conventional AJCC-TNM staging system, our model showed superior performance in predicting postoperative PFS for ICC patients. This tool not only provides a reliable basis for clinical risk stratification but also opens new avenues for treatment monitoring and personalized therapeutic decision-making, representing a potentially valuable clinical instrument for individualized prognosis assessment in ICC management.

## Data Availability

The datasets used or analyzed during the current study are available from the corresponding author on reasonable request. In order to protect study participants’ privacy, our data cannot be shared openly. Requests to access these datasets should be directed to LP, 836533071@qq.com.
