# Correction: A blood test-based nomogram to predict the progression-free survival of patients with intrahepatic cholangiocarcinoma after surgical resection

**DOI:** 10.3389/fonc.2026.2008653

**Published:** 2026-10-13

**Authors:** Lirong Peng, Yang Shi, Shuang Yang, Cunyan Li

**Affiliations:** 1 Department of Clinical Laboratory, The First Affiliated Hospital of Hunan Normal University, Changsha, China; 2 Department of Research and Development, Human Stem Cell National Engineering Research Center, Changsha, China

**Keywords:** intrahepatic cholangiocarcinoma, progression free survival, nomogram, blood biomarkers, prognostic model

An incorrect number was provided for 20231161. The correct number is W20243181.

The original version of this article has been updated.

