# Supplementary material for: A blood test-based nomogram to predict the progression-free survival of patients with intrahepatic cholangiocarcinoma after surgical resection
Source: Front Oncol. 2025 Jun 9;15:1507602. doi: 10.3389/fonc.2025.1507602 (PMC12183019; doi:10.3389/fonc.2025.1507602)
Supplement: Supplementary file 1 [file DataSheet1.docx]

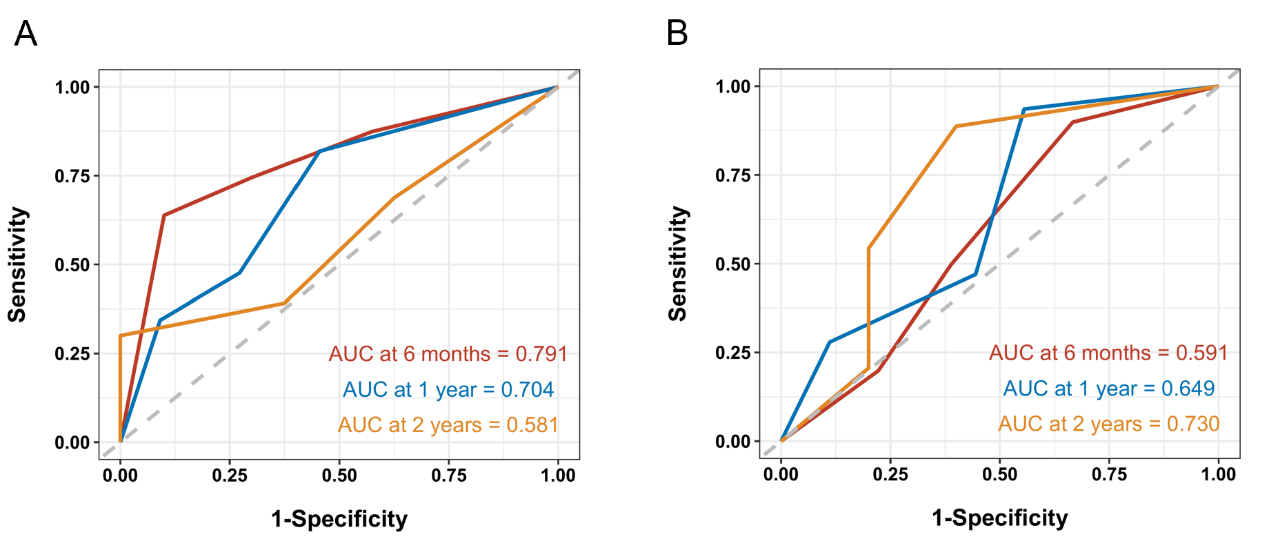
 Supplementary Figure 1. ROC curves of AJCC-TNM staging system in patients with ICC after surgical resection for half-year, 1-year, and 2-year PFS in the training (A) and validation (B) set.
